# Exercise as a Metabolic Regulator: Targeting AMPK/mTOR-Autophagy Crosstalk to Counteract Sarcopenic Obesity

**DOI:** 10.14336/AD.2025.0419

**Published:** 2026-06-05

**Authors:** Daoqi Zhang, Congfei Lu, Kai Sang

**Affiliations:** ^1^School of Physical Education, Xinyang Normal University, Xinyang 464000, China.; ^2^Physical Education and Sport Science, Fujian Normal University, Fuzhou 350117, China.; ^3^Biomedical Research Center of South China, Fujian Normal University, Fuzhou 350117, China.

**Keywords:** Sarcopenia obesity, AMPK, mTOR, Autophagy, Exercise

## Abstract

Sarcopenic obesity (SO), a geriatric syndrome characterized by the coexistence of progressive skeletal muscle atrophy and excessive adipose tissue accumulation, represents a growing public health challenge associated with aging populations. While multifactorial pathogenesis involves chronic inflammation, hormonal changes, and mitochondrial dysfunction, sedentary lifestyles and aging remain primary modifiable and non-modifiable risk factors, respectively. Mechanistically, exercise exerts dual therapeutic effects: (1) hypertrophy of type II muscle fibers through IGF-1/Akt/mTORC1 signaling activation, and (2) enhanced lipid β-oxidation via AMPK/PGC1α axis stimulation, thereby mitigating both sarcopenia and adiposity. The autophagy-lysosome system, a conserved cellular quality-control mechanism, orchestrates organelle turnover and nutrient recycling through three distinct pathways: macroautophagic, chaperone-mediated autophagy, and mitophagy. In SO, impaired proteolytic and lipolytic processes converge to induce autophagic flux blockade, manifested by accumulated p62/SQSTM1 and reduced LC3-II/LC3-I ratio. Targeting the AMPK/mTOR signaling nexus, which senses cellular energy status, emerges as a strategic intervention. Exercise-mediated ATP depletion activates AMPK while suppressing mTORC1, thereby synchronously inducing autophagy initiation (ULK1 phosphorylation) and lysosomal biogenesis (TFEB nuclear translocation). This metabolic reprogramming ultimately restores proteostasis and lipid homeostasis in myocytes and adipocytes.

## 1. Introduction

Aging leads to declines in muscle strength, mass, and physiological function, accompanied by increased fat accumulation and lipid deposition in non-adipose tissues. When this condition progresses, it develops into sarcopenic obesity (SO), an age-related obesity disorder characterized by abnormal age-dependent muscle atrophy and excessive body fat accumulation [1, 2, 3]. SO increases risks of falls, unsteady gait, balance disorders, and fractures, while exacerbating the health impacts of chronic diseases in older adults such as diabetes, cardiovascular/cerebrovascular diseases, and liver disorders [4, 5]. It has become a significant health risk for the elderly population, particularly in aging societies, with current prevalence rates ranging from 0.26% to 9.1% [6]. Incidence rises sharply with age, making individuals aged 80+ the highest-risk group, underscoring the urgent need for effective prevention and treatment strategies.

As an emerging disease concept, the underlying pathological mechanisms of SO remain incompletely understood [7-9]. Current evidence suggests that key factors include imbalances between protein synthesis/degradation, fat accumulation, and dysregulation of multiple signaling pathways. Consequently, modulating autophagy—a cellular process that clears dysfunctional organelles in aging muscle—has emerged as a critical therapeutic target for SO and a focus for elucidating its molecular mechanisms. While exercise interventions can regulate cellular autophagy, the specific role of exercise-mediated autophagy in ameliorating SO remains unclear. The AMP-activated protein kinase (AMPK) signaling pathway plays a central role in metabolic homeostasis and autophagy activation across tissues. We hypothesize that AMPK-mediated autophagy serves as a core therapeutic axis by which exercise simultaneously improves muscle and metabolic function in sarcopenic obesity. This review examines the role and molecular mechanisms of exercise-induced autophagy via the AMPK/mTOR signaling pathway in improving SO, offering novel insights into autophagy's therapeutic potential in exercise-based SO interventions.

## 2. Biological Pathways Underlying Sarcopenic Obesity

SO arises from the complex interplay between muscle loss and adipose tissue expansion, often exacerbated by aging-related hormonal decline, chronic low-grade inflammation, and metabolic dysregulation [10]. Clarifying their relationships is critical for understanding and treating SO.

### 2.1. Muscle-Fat Crosstalk and Ectopic Lipid Accumulation

SO involves a maladaptive interaction between degenerating skeletal muscle and expanding adipose tissue [11]. One hallmark is myosteatosis, characterized by the ectopic accumulation of lipids within and around skeletal muscle fibers; this not only mechanically disrupts contractile function but also contributes to local insulin resistance and mitochondrial dysfunction [12, 13], adipocytes secrete pro-inflammatory adipokines such as TNF-α, IL-6, and resistin, which infiltrate muscle tissue and activate catabolic pathways (e.g., NF-κB and STAT3) that promote proteolysis and inhibit myogenesis [14]. Furthermore, impaired β-oxidation in aging muscle increases the storage of lipid intermediates (e.g., ceramides, DAGs), which interfere with insulin signaling (via IRS1/PI3K/Akt) and disrupt protein synthesis [15-17]. Animal studies have shown that lipid overload in muscle cells leads to decreased PGC-1α expression and altered mitochondrial biogenesis [18]. In older adults with SO, magnetic resonance spectroscopy reveals elevated intramyocellular lipid (IMCL) that correlates with reduced strength and VO_2_max [19-21]. These changes indicate that muscle-fat crosstalk initiates a vicious metabolic cycle that accelerates both sarcopenia and obesity.

### 2.2 Hormonal Dysregulation and Anabolic Resistance

Hormonal changes with aging—particularly reductions in testosterone, growth hormone (GH), IGF-1, and estrogen—profoundly affect muscle metabolism and fat distribution [22]. Testosterone and IGF-1 promote protein synthesis via Akt/mTOR signaling, while estrogen enhances mitochondrial function and autophagy [1, 23-25]. In males, testosterone deficiency has been linked to type II fiber atrophy and increased fat mass [26, 27]. In females, menopause-associated estrogen loss results in both sarcopenia and visceral obesity, mediated in part through dysregulated AMPK/PGC-1α signaling and reduced antioxidant capacity [28]. Importantly, emerging data suggest sex-based differences in autophagy responsiveness to exercise and caloric stress. For instance, preclinical studies show that estrogen deficiency blunts exercise-induced LC3-II accumulation and TFEB nuclear translocation, while estrogen replacement partially restores this response [29, 30]. However, few human studies stratify outcomes by sex, highlighting the need for sex-specific clinical trials investigating anabolic resistance and autophagy modulation in SO.

### 2.3 Chronic Inflammation, Insulin Resistance, and Mitochondrial Dysfunction

SO is frequently associated with chronic low-grade systemic inflammation (inflammaging), characterized by elevated levels of IL-6, CRP, and TNF-α, which impair muscle regeneration and promote catabolism [31]. These cytokines inhibit insulin signaling through serine phosphorylation of IRS-1, suppressing Akt/mTORC1 activation and protein synthesis [32]. At the same time, inflammation activates proteolytic pathways such as ubiquitin-proteasome system (UPS) and autophagy in a maladaptive manner, resulting in net muscle loss [33]. Moreover, aging muscle exhibits mitochondrial DNA mutations, ROS overproduction, and diminished mitophagy [34, 35]. This mitochondrial dysfunction further amplifies insulin resistance, reduces ATP availability, and activates FoxO3a-mediated transcription of atrophy-related genes (e.g., Atrogin-1, MuRF1) [36]. These interconnected inflammatory and metabolic changes create a bioenergetic bottleneck that accelerates the progression of SO.

### 2.4 Comorbidities and Functional Heterogeneity in SO

SO rarely exists in isolation and is commonly complicated by type 2 diabetes, cardiovascular disease (CVD), osteoarthritis, and cognitive decline, which affect both its pathophysiology and response to treatment. In diabetic individuals, hyperglycemia and accumulation of advanced glycation end-products (AGEs) increase oxidative stress and suppress satellite cell function [37, 38]. Diabetic myopathy is also associated with reduced autophagic flux and impaired AMPK responsiveness, diminishing the muscle’s adaptive capacity to exercise [38]. Cardiovascular disease contributes indirectly through reduced physical activity, endothelial dysfunction, and systemic inflammation [39]. Osteoarthritis limits mobility, leading to muscle disuse and fat gain, cognitive impairment increases frailty risk and limits adherence to exercise programs [40]. Frailty, polypharmacy, and sarcopenia interact bidirectionally, creating a complex geriatric syndrome that is difficult to reverse. These comorbidities introduce heterogeneity into clinical SO populations, demanding individualized assessment and intervention strategies.

### 2.5 Methodological Limitations in SO Research

Despite the growing understanding of SO mechanisms, many cited studies rely on animal models or in vitro systems, which may not fully replicate the hormonal and inflammatory environment of aging humans. For example, rodent aging models often lack visceral obesity and the multimorbidity typical of older adults [41, 42]. Furthermore, the use of cross-sectional designs in human studies makes it difficult to establish causal links between mechanisms (e.g., autophagy impairment) and clinical outcomes (e.g., frailty, falls). Biomarker assessment also varies considerably. Many studies use static markers like LC3-II/LC3-I ratio or p62 levels, which do not accurately reflect autophagic flux [43]. Additionally, the sensitivity and specificity of noninvasive assessments of muscle quality and fat infiltration (e.g., ultrasound, bioimpedance) are limited. There is a pressing need for longitudinal, multi-omics, and muscle biopsy-based studies to better understand the progression and heterogeneity of SO in real-world populations.

In summary, sarcopenic obesity is driven by an intricate web of metabolic, hormonal, and inflammatory alterations, compounded by age, sex, and comorbid conditions. Understanding these mechanisms within clinically diverse populations is essential for developing personalized interventions that target both the sarcopenic and obese phenotypes.

**Figure 1.**
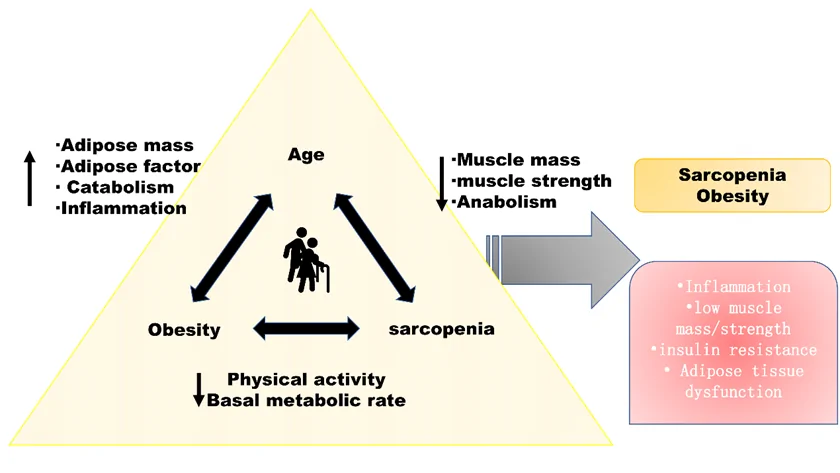
The “Vicious-Triangle” cycle ranging from aging, sarcopenia, to obesity might cause sarcopenic obesity.

## 3. Autophagy and Sarcopenic obesity (SO)

Autophagy is a catabolic process essential for the degradation of damaged proteins, dysfunctional organelles, and excess lipids. It plays a central role in cellular quality control and energy homeostasis, both of which are impaired in sarcopenic obesity (SO).

### 3.1 Role of Autophagy in Muscle and Adipose Tissue

In skeletal muscle, basal autophagy maintains proteostasis and mitochondrial quality [44]. Age-related suppression of autophagy leads to the accumulation of dysfunctional mitochondria and protein aggregates, impairing muscle regeneration and strength [45]. Markers such as the LC3-II/LC3-I ratio, p62 accumulation, and Beclin-1 expression reflect alterations in autophagic flux, with multiple studies reporting their decline in aging muscle [46]. Meanwhile, in adipose tissue, autophagy regulates lipid droplet turnover through “lipophagy” [47]. Its impairment results in excessive triglyceride storage, adipocyte hypertrophy, and chronic inflammation—hallmarks of SO.

### 3.2 Experimental and Clinical Evidence

Animal studies have confirmed that muscle-specific deletion of autophagy genes (e.g., Atg5, Atg7) results in severe myopathy and metabolic imbalance [48]. Similarly, inhibition of autophagy in adipose tissue impairs lipid mobilization and promotes insulin resistance. While these findings establish causality in preclinical models, evidence in humans remains largely correlative and mechanistically underexplored [49]. Limited muscle biopsy studies in elderly individuals have shown reduced LC3-II and increased p62, but variability in assay methods and sampling sites has hindered standardization [50]. To date, few studies have assessed autophagy biomarkers in SO patients specifically. Muscle biopsies and circulating LC3-II/p62 levels have been used inconsistently, and dynamic flux assays remain rare. These methodological limitations highlight the need for more comprehensive, standardized, and dynamic assessments of autophagy in human SO populations.

### 3.3 Methodological Gaps and Future Directions

Most mechanistic data on autophagy in SO derive from rodent models, which do not fully replicate the metabolic complexity, hormonal variability, or comorbidity burden of aging humans [51-53]. In addition, commonly used static markers (e.g., LC3-II/LC3-I ratio) do not accurately capture dynamic autophagy flux. Future research should prioritize: use of validated human muscle and adipose tissue samples; standardized protocols for autophagy biomarker quantification; longitudinal studies linking autophagy modulation to functional outcomes in SO.

In summary, impaired autophagy contributes to both the sarcopenic and obese features of SO. Although preclinical studies offer valuable mechanistic insights, human-specific data and dynamic assessments are essential for validating autophagy as a therapeutic target.

## 4. AMPK/mTOR Pathway as a Central Regulator of Autophagy

AMPK and the mechanistic target of rapamycin complex 1 (mTORC1) constitute a central nutrient-sensing axis that regulates autophagy initiation. Their opposing actions—AMPK activating and mTORC1 suppressing autophagy—integrate cellular energy status with catabolic responses.

### 4.1 Dual Regulation of Autophagy via AMPK and mTORC1

Under low-energy conditions (e.g., increased AMP/ATP ratio), AMPK becomes activated and phosphorylates key autophagy initiators such as ULK1 at Ser555 and Ser317 [54, 55]. This activation is reinforced by AMPK-mediated inhibition of mTORC1, which normally phosphorylates ULK1 at multiple sites, including Ser555 and Ser317 to prevent autophagy [56]. AMPK also targets TSC2 and Raptor to suppress mTORC1 activity and facilitates TFEB nuclear translocation, thereby upregulating lysosomal and autophagy-related genes [57, 58]. In contrast, mTORC1 becomes activated in response to growth factors, amino acids, or insulin, inhibiting autophagy and promoting protein synthesis. Persistent mTORC1 activity in obesity and aging may impair cellular quality control by suppressing autophagy despite accumulated metabolic stress.

### 4.2 Evidence from Experimental Models

Animal models and cell culture studies consistently support the role of AMPK/mTOR signaling in regulating autophagy across multiple tissues [59]. For example, rapamycin-induced mTORC1 inhibition enhances autophagic flux and ameliorates lipid accumulation in hepatocytes, while AMPK activators improve mitochondrial quality in skeletal muscle [60]. However, few studies have demonstrated direct modulation of this pathway in older adults with SO. Existing data are mostly extrapolated from isolated cell studies or young rodent models.

### 4.3 Translational Implications and Limitations

Despite the mechanistic clarity in vitro and in vivo, translation of these findings into human interventions remains limited [61]. The context-dependent behavior of AMPK and mTOR—modulated by age, nutritional status, comorbidities, and medication use—complicates direct clinical targeting [55, 62]. Furthermore, pharmacological agents like metformin and rapamycin activate AMPK or inhibit mTOR [63], but their long-term effects on functional outcomes in SO are unclear and may carry adverse effects. Understanding how modifiable behaviors like exercise influence the AMPK/mTOR axis may yield actionable targets for community-level prevention strategies.

Future studies should examine how lifestyle interventions such as exercise or caloric restriction modulate AMPK/mTOR-autophagy signaling in SO patients, using both molecular biomarkers and clinical endpoints.

## 5. Impact of Exercise Training on SO

Exercise is a primary non-pharmacological intervention recommended for the prevention and management of sarcopenic obesity (SO). It improves muscle strength, promotes fat utilization, and activates autophagy-related pathways such as AMPK/mTOR. However, exercise efficacy is influenced by training type, intensity, duration, and inter-individual differences. Below, we compare the effects of aerobic, resistance, and combined exercise modalities on SO parameters (Table 1).

**Table 1:** Effects of different exercise modalities on body composition in SO patients.

Sample Size	Mean Age	Outcome Measures	Intervention	Intervention Outcomes
**28**	72	BMI & HGS	10 weeks RE (2 sessions/week) vs. no exercise	Muscle mass (kg) Significant difference observed
**35**	>60	BF% & SMI	12 weeks RE (3 sessions/week) vs. no exercise	Fat mass (kg)-0.58 Skeletal muscle index significant decline
**46**	67	BF% & SMI	12 weeks RE vs. no exercise	Skeletal muscle mass (kg) +0.73 (95% CI) Muscle mass (kg)+2.63 (95% CI)
**60**	69	BMI & SMI	8 weeks RE, AE, RE + AE vs. no exercise	Fat mass (kg):RE: -1, AE: -0.7, RE + EA: -1.1, Skeletal muscle mass(kg): RE: +0.1, AE: +0.1, RE + EA: +0.2, Grip strength (kg): RE: +3.5, AE and RE + EA: No significant difference
**133**	67	BMI & PT	24 weeks RE (3 sessions/week) vs. no exercise	Skeletal muscle mass and strength Significant increase
**139**	81	BF%, SMI, & HGS	3 months AE + RE vs. no exercise	Arm muscle mass (kg): +1.8, p < 0.05; Leg muscle mass (kg): +2.2, p < 0.05; Strength (kg): +17.8%, p = 0.119

Abbreviations: BMI-Body Mass Index; FM- Fat Mass; SMM-Skeletal Muscle Mass; BF%-Body Fat Percentage; SMI-Skeletal Muscle Index; RE-Resistance Exercise; AE-Aerobic Exercise; HGS-Handgrip Strength; PT-Peak Strength; PPT-Physical Performance Test.

### 5.1 Effects of Different Exercise Modalities

#### 5.1.1 Aerobic Exercise

Endurance training enhances muscular endurance in aged skeletal muscle but has limited impact on strength or mass. Its benefits arise from mitochondrial biogenesis, improved cardiovascular function (e.g., increased stroke volume), and elevated capillary density, which collectively enhances muscle oxidative capacity. Additionally, aerobic exercise effectively controls fat mass, particularly when combined with dietary interventions, making it a robust anti-obesity strategy. In a study investigating aerobic exercise in SO populations [64], 8 weeks of endurance training in 60 adults aged 65-75 years significantly reduced body fat mass and visceral adipose tissue compared to controls, though skeletal muscle mass remained unchanged.

#### 5.1.2 Resistance Exercise

For SO patients, skeletal muscle strength—particularly in the lower limbs—is critical. Resistance training potently stimulates net muscle protein synthesis, inducing hypertrophy and improving muscle mass and strength, thus emerging as the optimal exercise modality for SO. A study of 1,328 adults over 50 years old demonstrated that 20 weeks of resistance training (2-3 sessions/week) increased skeletal muscle mass by an average of 1.1 kg [65]. Similarly, a 12-week elastic band training regimen in older women with SO significantly improved muscle mass, strength, and physical performance compared to non-exercising controls [66]. Recent findings also show that 8 weeks of resistance exercise in 60 elderly SO patients preserved skeletal muscle mass, reduced fat mass, and increased handgrip strength relative to sedentary peers [67].

#### 5.1.3 Combined Exercise

Emerging evidence highlights the synergistic benefits of integrating aerobic and resistance training. This approach concurrently addresses fat loss and muscle preservation, addressing SO’s dual pathology. Further research is needed to optimize training protocols for SO populations.

### 5.2 Variability in Exercise Response

Inter-individual variability significantly affects exercise outcomes in SO. Factors such as biological sex, age subgroup (e.g., "young-old" vs. "old-old"), hormonal status, and comorbidities (e.g., diabetes, cardiovascular disease) modulate training response. For instance, older women may exhibit reduced hypertrophic responses due to lower baseline anabolic hormone levels [68]. Similarly, patients with type 2 diabetes often demonstrate attenuated improvements in insulin sensitivity, potentially due to underlying mitochondrial dysfunction and chronic inflammation.

### 5.3 Potential Limitations and Risks

Although exercise has broad benefits, it is not universally effective or safe in all SO populations. Excessive intensity or volume may induce muscle damage, exacerbate joint stress, or provoke inflammatory rebound in frail individuals [69, 70]. Moreover, underlying conditions such as osteoporosis or cardiovascular disease may limit safe participation. Importantly, overtraining has been associated with elevated ROS levels and a temporary downregulation of autophagic activity in some experimental models [71].

### 5.4 Practical Recommendations

Current evidence supports the use of moderate-intensity CE (2-3 sessions/week of RE + 1-2 sessions/week of AE) in older adults with SO [70, 72]. Tailoring exercise prescriptions based on functional capacity, health status, and recovery ability is essential. Incorporating periodic reassessment and professional supervision can optimize benefits and reduce risks.

In summary, exercise represents a cornerstone therapy for SO, yet its implementation must consider biological variability and potential adverse effects. Future studies should aim to personalize exercise interventions using biomarker-guided approaches and assess long-term safety in high-risk populations.

## 6. Possible Mechanisms by Which Exercise Activates Autophagy via the AMPK/mTOR Signaling Pathway to Improve SO

### 6.1 Exercise-Induced Activation of Autophagy via the AMPK/mTOR Signaling Pathway

Exercise is widely recognized for its capacity to promote healthy aging and reduce the risk of age-related diseases. Among its molecular effects, the modulation of autophagy via the AMPK/mTOR signaling axis has gained considerable attention. Both preclinical and emerging human studies have shown that endurance exercise activates AMPK, which subsequently phosphorylates ULK1 and attenuates mTORC1-mediated inhibition of autophagy initiation [73-75]. Endurance exercise activates AMPK, phosphorylates ULK1, and reduces mTOR-mediated inhibitory phosphorylation of ULK1 [75]. For instance, in an ovariectomized rat model, an 8-week swimming intervention restored the expression of autophagy-related proteins (including mTOR, ULK1, Bcl-1, and Atg7), thereby mitigating estrogen-deficiency-induced muscle atrophy and autophagy impairment [76]. These findings suggest that exercise-mediated AMPK/mTOR signaling may activate autophagy. During exercise, muscle contraction consumes substantial ATP, increasing the AMP/ATP ratio and activating AMPK. Enhanced AMPK activity promotes autophagy through the following mechanisms: Direct regulation of ULK1: AMPK phosphorylates ULK1 at multiple residues (Ser467, Ser555, Thr574, Ser637), directly enhancing its activity [77]; Indirect regulation via mTORC1 inhibition: AMPK phosphorylates and activates TSC2, which suppresses mTORC1 [78, 79]. Additionally, AMPK inhibits mTORC1 by phosphorylating Raptor, a regulatory subunit of mTORC1 [56]. Thus, AMPK promotes autophagy both by directly activating ULK1 and by relieving mTORC1-mediated inhibition of ULK1. These findings delineate a coherent mechanistic pathway whereby exercise-induced energy stress activates AMPK, suppresses mTORC1, and ultimately enhances autophagic flux. However, most mechanistic insights are derived from rodent models, and large-scale human trials confirming these molecular events in sarcopenic obesity populations remain limited.

### 6.2 Exercise-Induced Autophagy Enhances Lipolysis and Attenuates Lipid Accumulation in Sarcopenic Obesity

Adipose tissue not only serves as an energy reservoir but also functions as a dynamic endocrine organ, secreting adipokines such as leptin and adiponectin to regulate systemic energy homeostasis. Dysregulation of lipid metabolism, particularly ectopic lipid accumulation in skeletal muscle and liver, is a hallmark of sarcopenic obesity (SO) [80]. Autophagy plays a pivotal role in lipid turnover through a process known as “lipophagy,” wherein intracellular lipid droplets are engulfed and degraded in autolysosomes. Early studies on lysosomal acid lipase (LAL) deficiency established that impaired autophagic flux leads to triglyceride (TG) and cholesterol accumulation [81, 82]. Under energy-rich conditions, both lipolysis and autophagy are downregulated, favoring lipogenesis. In contrast, during energy stress—such as exercise—autophagy is activated to hydrolyze TGs, thereby releasing free fatty acids (FFAs) for β-oxidation [83]. Pharmacological and genetic studies have further supported this mechanism. Induction of autophagy via agents like rapamycin reduces TG content and lipid droplet number [84]. whereas hepatic deletion of key autophagy genes (Atg7, Tfeb) exacerbates steatosis and obesity phenotypes [85]. Moreover, autophagy enhances β-adrenergic signaling, which promotes hormone-sensitive lipase-mediated lipolysis [86]. In the context of SO, reduced autophagic capacity contributes to adipocyte hypertrophy, impaired lipolysis, and intramuscular fat infiltration. Exercise-induced activation of AMPK/mTOR signaling restores autophagy, thereby promoting lipid clearance, suppressing lipogenesis, and improving adipose-muscle metabolic crosstalk. However, most evidence stems from hepatic or general obesity models, and the specific lipophagic response to exercise in human SO populations remain underexplored [87]. Future studies should aim to characterize tissue-specific lipid clearance pathways and their responsiveness to different exercise modalities in aging populations with SO.

### 6.3 Autophagy-Mediated Protein Turnover as a Defense Against Sarcopenia in SO

The maintenance of skeletal muscle mass relies on a delicate balance between protein synthesis and degradation. While the ubiquitin-proteasome system primarily degrades short-lived proteins, the autophagy-lysosomal pathway is essential for clearing long-lived proteins, misfolded aggregates, and damaged organelles—key to preserving proteostasis, especially during aging [88, 89]. In animal models, muscle-specific deletion of autophagy-related genes such as Atg5 or Atg7 results in severe proteotoxic stress, characterized by p62/SQSTM1 accumulation, mitochondrial dysfunction, and reduced contractile force [90]. Similarly, chronic activation of mTORC1 suppresses autophagy and promotes protein aggregation, leading to progressive myopathy [91]. With advancing age, autophagic flux diminishes, impairing both protein and organelle quality control. This decline has been implicated as a key mechanism underlying sarcopenia, the muscle loss component of SO [92]. Exercise, by activating AMPK and inhibiting mTORC1, reactivates autophagic pathways, facilitating the clearance of damaged cellular components. This restores proteostasis and helps maintain muscle fiber integrity and function [93]. While robust in preclinical studies, the extent to which exercise-induced autophagy improves proteostasis in sarcopenic human muscle remains insufficiently explored. Clinical investigations incorporating muscle biopsies and autophagy biomarkers (e.g., LC3-II/I ratio, p62 levels) before and after structured exercise interventions could clarify these translational mechanisms.

### 6.4 Autophagy Alleviates Anabolic Resistance and Supports Muscle Protein Homeostasis in SO

Anabolic resistance—the diminished ability of skeletal muscle to respond to anabolic stimuli such as amino acids, insulin, or mechanical loading—is a hallmark of sarcopenia and is exacerbated in sarcopenic obesity (SO) [94]. In this context, the pathological interplay between excessive adiposity and muscle tissue promotes a cycle of chronic inflammation, insulin resistance, and impaired protein synthesis. Age-related muscle loss facilitates intramuscular fat infiltration, while hypertrophied adipocytes and adipose-derived cytokines such as leptin and TNF-α amplify inflammatory signaling and suppress anabolic pathways [95]. Accumulated lipid intermediates (e.g., diacylglycerol, ceramides) disrupt insulin signaling by promoting serine phosphorylation of IRS-1, thereby reducing Akt/mTORC1-mediated protein synthesis [43, 96]. Free fatty acids and systemic insulin resistance further reinforce anabolic blunting in skeletal muscle [97]. Exercise alleviates anabolic resistance through multiple mechanisms: it reduces visceral and intramuscular fat, enhances insulin sensitivity, and suppresses chronic low-grade inflammation. Notably, autophagy has emerged as a key mediator of these benefits. Impaired autophagy correlates with elevated inflammasome activation and insulin resistance in aged muscle [98], while autophagy induction improves insulin signaling and reduces inflammatory cytokine production [99]. Therefore, AMPK/mTOR-mediated autophagy activation may restore anabolic sensitivity by resolving lipotoxicity, improving mitochondrial quality, and enabling more efficient nutrient signaling. Nevertheless, mechanistic studies specifically linking autophagy restoration to improved muscle protein synthesis in older SO populations are scarce [100]. Future work should explore whether enhancing autophagy can directly reverse anabolic resistance in human muscle under obese and inflamed conditions.

Exercise-induced energy stress increases the AMP/ATP ratio, leading to AMPK activation and mTORC1 inhibition. This dual regulation initiates autophagy via ULK1 phosphorylation and TFEB translocation. Enhanced autophagic flux facilitates the clearance of damaged mitochondria and protein aggregates in skeletal muscle, promotes lipid droplet degradation in adipose tissue, improves insulin sensitivity, and alleviates inflammation. These molecular effects jointly contribute to improved muscle mass, reduced fat accumulation, and reversal of anabolic resistance, ultimately mitigating the pathophysiology of SO.

**Figure 2.**
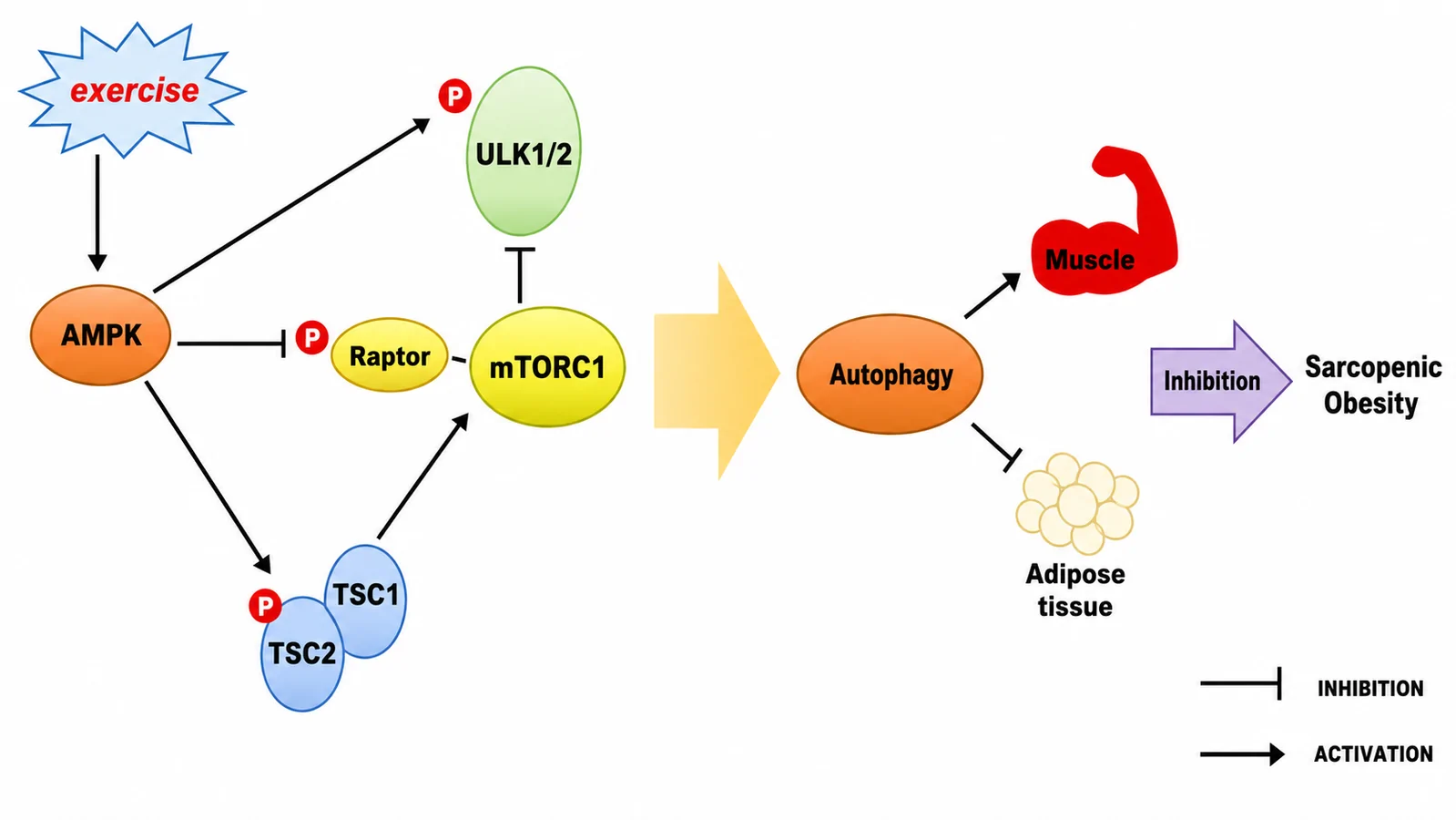
Schematic representation of how exercise activates autophagy via AMPK/mTOR signaling to ameliorate sarcopenic obesity (SO).

### 6.5 Summary of Mechanisms and Future Directions

Collectively, exercise-induced autophagy via the AMPK/mTOR axis improves sarcopenic obesity through multiple, converging mechanisms—enhancing lipid clearance, supporting protein turnover, and restoring anabolic sensitivity. However, the majority of evidence derives from animal studies or cellular models. Human trials incorporating molecular endpoints (e.g., autophagy flux markers) are needed to confirm these effects and refine personalized exercise prescriptions for elderly populations with SO.

## 7. Conclusions

Sarcopenic obesity (SO) represents a complex geriatric syndrome arising from intertwined pathologies of muscle degeneration and adipose tissue expansion. Mounting evidence suggests that exercise exerts beneficial effects on SO not only by increasing energy expenditure and improving physical function but also by reprogramming intracellular signaling networks. Activation of the AMPK/mTOR-autophagy axis has emerged as a central mechanism linking mechanical and metabolic stimuli to cellular quality control in muscle and adipose tissues.

Mechanistically, exercise-induced autophagy facilitates the removal of dysfunctional mitochondria and misfolded proteins, enhances lipolysis, suppresses ectopic lipid deposition, and improves insulin sensitivity. These processes work synergistically to restore proteostasis and metabolic flexibility, thereby counteracting both the sarcopenic and obese components of SO.

Despite these advances, most current knowledge is derived from preclinical models, and the molecular relevance of these pathways in elderly humans with SO remains underexplored. Clinical trials incorporating biopsies, circulating autophagy markers, and functional outcomes are urgently needed to validate mechanistic hypotheses and translate them into evidence-based interventions. Moreover, individual variability due to sex, comorbidities (e.g., diabetes, cardiovascular disease), and aging stage must be systematically addressed in future studies.

We propose an integrative model whereby exercise acts as a systemic metabolic regulator that activates autophagy via AMPK, inhibits mTORC1, and remodels both skeletal muscle and adipose tissue toward a healthier phenotype. This conceptual framework connects molecular mechanisms to population-level interventions and opens new avenues for designing targeted exercise prescriptions.

Future research should prioritize clinical validation, biomarker-guided prescriptions, and long-term outcome trials to close the gap between molecular evidence and public health impact. Priority directions include: (1) longitudinal trials validating autophagy modulation in SO; (2) biomarker-guided personalization of exercise; (3) integration of comorbidity-adjusted protocols in clinical settings.
